# DLUT: Decoupled Learning-Based Unsupervised Tracker

**DOI:** 10.3390/s24010083

**Published:** 2023-12-23

**Authors:** Zhengjun Xu, Detian Huang, Xiaoqian Huang, Jiaxun Song, Hang Liu

**Affiliations:** 1 School of Engineering, Huaqiao University, Quanzhou 362021, China; 21014084015@stu.hqu.edu.cn (Z.X.); 22014084017@stu.hqu.edu.cn (J.S.); 22013084001@stu.hqu.edu.cn (H.L.); 2 School of Information Science and Engineering, Huaqiao University, Xiamen 361021, China; 21013082022@stu.hqu.edu.cn

**Keywords:** deep learning, object tracking, unsupervised learning, decoupled learning, pseudo-labels

## Abstract

Unsupervised learning has shown immense potential in object tracking, where accurate classification and regression are crucial for unsupervised trackers. However, the classification and regression branches of most unsupervised trackers calculate object similarities by sharing cross-correlation modules. This leads to high coupling between different branches, thus hindering the network performance. To address the above issue, we propose a Decoupled Learning-based Unsupervised Tracker (DLUT). Specifically, we separate the training pipelines of different branches to unlock their inherent learning potential so that different branches can fully explore the focused feature regions of interest. Furthermore, we design independent adaptive decoupling-correlation modules according to the characteristics of each branch to obtain more discriminative and easily locatable feature response maps. Finally, to suppress the noise interference brought by unsupervised pseudo-label training and highlight the foreground object, we propose a novel suppression-ranking-based unsupervised training strategy. Extensive experiments demonstrate that our DLUT outperforms state-of-the-art unsupervised trackers.

## 1. Introduction

As one of the fundamental research topics in computer vision, visual object tracking holds extensive applicability in areas such as autonomous driving, video surveillance, unmanned aerial vehicles, and military applications [1]. In recent years, significant progress has been made in object tracking, especially with the advent of deep learning-based trackers. However, existing deep learning-based trackers need to learn rich feature representations from a large number of supervised training datasets with labels. With the rapid growth of the Internet, the number of online videos is increasing dramatically. Most of these unlabeled videos could also be used as training sets for visual tracking. Therefore, how to effectively utilize unlabeled videos has become one of the future research focuses in object tracking.

A tracker trained in an unsupervised manner on unlabeled datasets is referred to as an unsupervised tracker. It validates tracking by exploiting rich self-supervision signals from video sequences. For example, UDT [2] acquires sufficient supervision signals during forward tracking and backward validation, prompting unsupervised tracking and laying the foundation for subsequent research. Existing unsupervised trackers can be broadly classified into two categories. The first category constructs single video frames as template-search frame pairs, making them susceptible to interference from similar objects or incapable of adapting to significant appearance changes during tracking. Although these trackers offer fast tracking speeds, they often lack stability. For instance, such trained trackers cannot adapt to objects with pronounced appearance changes. The second category involves improving existing tracking frameworks by incorporating an additional regression branch to effectively utilize temporal information about object variations over time, so as to capture stronger motions and appearance changes, and thus significantly increase the tracking robustness. Hence, the latter has been garnering increasing attention. The representative USOT [3] utilized a single-frame pair to train a naive Siamese tracker. During the inference stage, the result is served as the output of the offline branch and updated through online training with a cyclic memory queue during periodic training. The final tracking outcome is jointly determined by both the offline and online branches. The latest ULAST [4] method, also belonging to the second category, further explores the temporal correspondence between classification and regression branches. However, our research has uncovered a significant issue with the second category of trackers: existing trackers tend to have their classification and regression branches sharing common cross-correlation modules, which may lead to inherent attention conflicts between different task branches in actual tracking scenarios.

During the training process, the classification and regression branches exhibit differences in the focused target feature regions. Among them, the former mainly focuses on the salient feature regions of the target to obtain rich classification information, while the latter focuses on the edge features of the target to predict the accurate target position. The inconsistent tasks of the two branches lead to conflicts in task attention points. In the area of object detection, IoU-Net [5] and Double-Head RCNN [6] have made initial explorations to address such issues. The latter decouples the sibling head and uses two branches for classification and localization, respectively. Although double-head RCNN mitigates parameter sharing and reduced coupling associations to some extent, the inherent conflicts are not completely resolved, as both branches still rely on the same proposal generation for their input features. Subsequently, TSD [7] further addresses the conflicts by introducing a task-aware spatial decoupling operator. In the field of object tracking, such conflicts can potentially lead to compromised rather than optimal tracking results, as trackers attempt to balance classification and regression scores during training. Thus, existing trackers typically avoid using the sibling head in their classification/regression branches, as exemplified by the classic tracker Ocean [8]. However, both branches still share the same cross-correlation modules for their input features while neglecting task specificity, and thus still fail to handle the conflicts effectively. To address such issues, we further explore the task-specific network architecture and achieve superior tracking performance.

**Figure 1:**
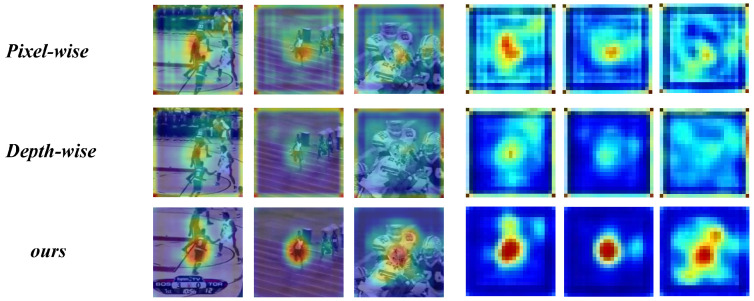
Comparison of the proposed decoupling-correlation operation of our DLUT with the commonly used Pixel-wise and Depth-wise on three challenging sequences from OTB100. Our decoupling-correlation operation accurately recognizes objects in a pseudo-label training environment, even when affected by similar objects, motion blur, and object occlusion. In contrast, Pixel-wise and Depth-wise are difficult to adapt to and are easily disturbed by complicated backgrounds.

In our study, we focus on the decoupled structure for unsupervised trackers. By designing adaptive decoupling-correlation modules, we further eliminate the coupling between different branches, thus fully releasing the learning capacity of unsupervised trackers. To achieve deep decoupling between different branches, we propose a decoupled learning-based unsupervised tracker, called DLUT. Figure 1 presents a comparison of heatmap visualizations of our method and classic correlation modules. Specifically, our DLUT consists of both offline and online modules. The former includes the classification and regression branches, while the latter includes the online queue branch. By decoupling the training pipelines of these three branches, each branch can be trained independently for optimal training results. Furthermore, inspired by AutoMatch [9], we design three different learning modules according to the characteristics of each branch. It is worth noting that the parameters of these modules are designed as learnable parameters to allow each module to be trained adaptively and independently, and to better adapt to different task goals. Finally, to overcome the challenges faced by unsupervised training strategies, such as numerous uninformative samples, unbalanced distribution of positive and negative samples, and inaccurate pseudo-labels, we devise a loss function based on a Suppression-Ranking Strategy (SRS). This designed loss function reduces the background noise while sorting the foreground and background samples, thus highlighting the focused foreground object and improving the tracking performance.

To validate the effectiveness of the proposed DLUT, we conduct evaluations on prominent benchmark datasets and compare them with state-of-the-art unsupervised and supervised trackers. The primary contributions of our work are summarized as follows:

- We propose a decoupled learning-based unsupervised tracker (DLUT). By decoupling the training pipelines of different branches, we fully unleash the unsupervised learning capability of the DLUT.
- Considering the characteristics of each branch and in line with the proposed decoupled framework, we devise three independent decoupling-correlation modules. These modules are able to adapt to the goals of different branches and realize independent and adaptive training without disrupting the respective training pipelines.
- We devise an unsupervised training strategy based on suppression ranking, which is able to suppress background noise and sort foreground and background samples to highlight the foreground object.

## 2. Related Work

### 2.1. Supervised Tracking

Benefiting from recent advances in deep learning, Siamese network-based trackers have made tremendous progress. As the pioneering work, SiamFC [10] transforms the tracking problem into a matching one, in which feature extraction is performed on template and search frames by using a shared-weight backbone network, and then the response maps are generated to locate the object with cross-correlation modules. Building on SiamFC, SiamRPN [11] introduces the Region Proposal Network (RPN) [12] in the object recognition domain, which generates candidate boxes with different scales to tackle the severe deformation challenge. Dsiam [13] employs object deformation transformation and background suppression transformation mechanisms to increase the tracking robustness, while CLNet [14] and MemTrack [15] exploit contextual information and spatiotemporal relationship modeling to improve the tracking performance. On the other hand, anchor-free tracking networks exhibit powerful potential. As a representative example, Ocean [8] exploits feature alignment to address irregular and unstable object shape problems.

In recent years, transformer-based [16] models such as [17,18,19] have achieved state-of-the-art performance in visual tracking. Such models, based on the two-stream two-stage architecture, utilize a transformer for feature extraction and fusion to achieve object tracking with high accuracy and low computation. [20,21,22,23,24,25,26] under the one-stream one-stage architecture process template and search regions within the same transformer. This efficient feature fusion has led to a significant improvement in the tracking performance. The latest tracker MAT [27] uses two identical decoders to reconstruct the target region. Considering the characteristics of the tracking task, the ability of the transformer to handle sequence information is adjusted to optimize the tracking performance [28,29]. It should be noted that all the above trackers are supervised trackers, which require a significant amount of labeled video sequences. In contrast, unsupervised trackers can be trained by extracting self-supervised signals from the samples, eliminating extensive labeling costs.

### 2.2. Unsupervised Tracking

The pioneer UDT [2] is the first unsupervised deep tracker based on the Discriminative Correlation Filter (DCF) [30], which trains the tracker by calculating the consistency loss between two tracking trajectories. The superior performance obtained by UDT reveals the feasibility of exploiting the consistency property of video objects to train the tracker. This provides new insights for the study of unsupervised trackers. CycleSiam [31] extends the concept of consistency learning to Siamese network structures, while JSLTC [32] models video frames by computing an inter-frame affinity matrix and uses the obtained correlations for tracking. S2SiamFC [33] focuses on spatial monitoring and utilizes static frames to create training pairs. On the other hand, PUL [34] introduces contrastive learning to build a more discriminative model and uses robust loss functions during training to explore temporal correspondence blocks. USOT [3] employs optical flow to extract pseudo-labels and trains a naive Siamese tracker by constructing static single-frame pairs followed by iterative training over a larger time span. Recently, ULAST [4] introduces a novel unsupervised tracking framework that exploits abundant temporal self-supervision signals to learn temporal correspondences on the classification and regression branches, resulting in significant performance improvements. GOT [35] integrates three prediction branches and presents a lightweight and high-performance unsupervised tracker without offline pre-training. Based on the above unsupervised trackers, we revisit the unsupervised tracking pipeline and identify certain limitations in existing tracking structures. In addition, Siamese network-based tracking frameworks are sensitive to the selected matching operator, and the pseudo-labels used in unsupervised learning are often contaminated with substantial background noise interference. These problems hinder the progress of unsupervised trackers. To tackle these challenges, we propose a decoupled learning-based unsupervised tracker (DLUT) and achieve superior tracking performance.

### 2.3. Decoupled Structures

Decoupled structures are first applied to object detection. Led by pioneering studies such as the R-CNN family [12,36,37] and Feature Pyramid Networks (FPNs) [38], significant progress has been made in object detection. However, for the most common object classification and detection tasks, there has been an increasing focus on classification and localization predictions while overlooking their inherent correlations. This leads to performance limitations due to certain hidden bottlenecks. Subsequently, some studies have started to recognize this problem, that is, there exists an inherent conflict between different branches. IoU-Net [5] first revealed this conflict, where features with high classification scores do not usually match the quality of their bounding boxes. To solve this problem, IoU-Net introduces an additional IoU prediction. Double-head RCNN [6] attempts to mitigate parameter sharing between tasks by decoupling sibling heads and replacing them with separate classification and localization branches. However, this inherent conflict is not fully resolved since the features provided to both classification and localization branches are generated from the same proposed ROI pool and the input features to the branches remain identical. Subsequently, TSD [7] presents a task-aware spatial disentanglement operator that generates task-specific feature representations by proposing estimation and detection heads. This approach eliminates the constraints between classification and localization, thus achieving deep decoupling for both tasks. Unlike the aforementioned studies, we investigate an adaptive decoupling structure according to the characteristics of different branches of the unsupervised tracker. The proposed decoupling framework and cross-correlation modules facilitate different branches to be adequately trained, which activates the potential of the unsupervised tracker effectively.

## 3. Proposed Method

### 3.1. Preliminary

Our DLUT is built upon a Siamese network-based [10] tracker. Siamese networks model the relationship between template images and search images by using naive cross-correlation operations to learn feature similarity for tracking objects in video frames, ultimately achieving target classification and regression. Therefore, appropriate cross-correlation operations are of paramount importance for Siamese networks.

The Siamese network consists of two branches: one for the initial template *z* and the other for the search image *x*. Both branches share the feature extraction network φ(·) with weight parameters θ. Each branch obtains the template feature $\bar{z}=\varphi \left(z,\theta \right)\in R^{C\times H_{z}\times W_{z}}$ and the search feature $\bar{x}=\varphi \left(x,\theta \right)\in R^{C\times H_{x}\times W_{x}}$ via the feature extraction network, respectively, and then calculates response map $C=g\left(\bar{z},\bar{x}\right)=\varphi \left(z,\theta \right)*\varphi \left(x,\theta \right)$ for $\bar{z}$ and $\bar{x}$ via cross-correlation operations g(·). Each element in the response map *C* corresponds to the similarity between the initial template *z* and subregions of the search image *x*. Furthermore, the position with the highest response value in *C* is more likely to be the center of the object. The fundamental process of template matching in the Siamese network can be described as follows:

(1) $$\begin{array}{c} C=g\left(\bar{z},\bar{x}\right)=\varphi \left(z,\theta \right)*\varphi \left(x,\theta \right) \end{array}$$

where θ represents the shared weight parameters and ∗ denotes the cross-correlation operation.

**Figure 2:**
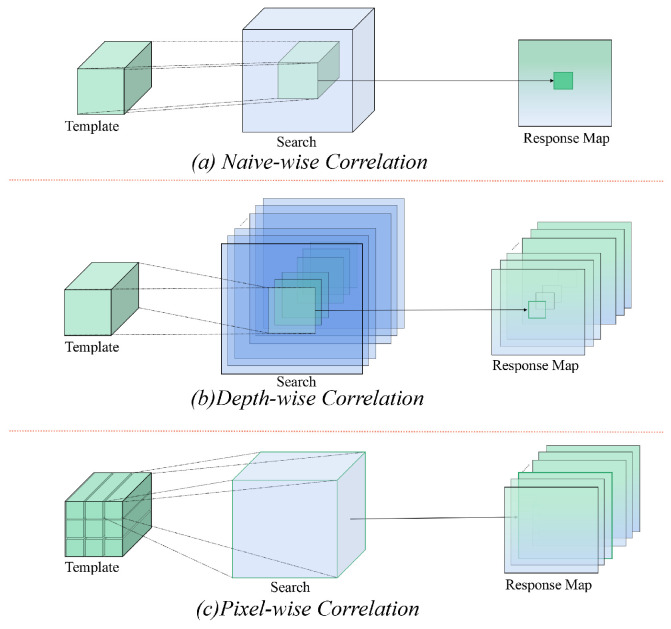
Comparison of different cross-correlation operations. From top to bottom, these figures are (**a**) Naive-wise cross-correlation proposed in SiamFC, (**b**) Depth-wise cross-correlation proposed in SiamRPN++, and (**c**) Pixel-wise cross-correlation proposed in Alpha-Refine.

As shown in Figure 2, early SiamFC utilizes a naive cross-correlation operation to implement template matching in the Siamese network. Specifically, this cross-correlation operation involves a simple convolution operation where template features slide over the search features to compute inner products, resulting in a single-channel response map. Subsequent methods like SiamRPN++ [39] and Alpha-Refine [40] exploit optimized cross-correlation modules, such as Depth-wise and Pixel-wise, to reduce the number of parameters and improve matching performance. Inspired by AutoMatch [9], we propose independent adaptive cross-correlation modules for the decoupled framework. These modules leverage the advantages of Depth-wise and Pixel-wise and adaptively learn to achieve comprehensive training for different branches according to the characteristics of each branch.

### 3.2. Unsupervised Tracking Network Based on Decoupled Framework

#### 3.2.1. Network Architecture

**Figure 3:**
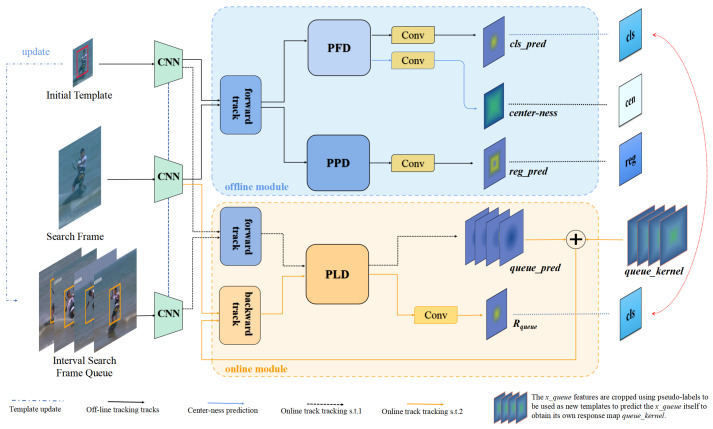
NetworkArchitecture. Our decoupled learning-based unsupervised tracker (DLUT) consists of offline and online modules. Specifically, the offline module generates classification response maps *cls_pred*, regression response maps *reg_pred*, and centerness response maps *center-ness*. On the other hand, the online module first predicts the response map *queue_pred* between the template and interval search queue, subsequently updates the template with features cropped by pseudo-labels, and then predicts the response map of the interval search queue *queue_kernel*. Finally, the features of *queue_pred*, *queue_kernel*, and search region features are aggregated to generate the final response map $R_{queue}$. For more details on the data flow, please refer to Section 3.5.

The network architecture of the proposed decoupled learning-based unsupervised tracker (DLUT) is depicted in Figure 3. Our DLUT contains an offline module and an online module, where the former is used to capture the object’s appearance and locate the target object, while the latter is used to capture time-varying object features for robust tracking. On the left side of the network architecture, the input is composed of the initial template frame, the search frame, and an interval search frame queue. Essentially, the interval search frame queue consists of search frames sampled at specific intervals within the same video sequence for training the online branch. Moreover, ResNet50 [41] is used to perform feature extraction. Unlike existing tracking frameworks, the proposed decoupled framework splits the tracking data flow into three branches, i.e., classification branch, regression branch, and online queue branch, according to the characteristics of different tasks. Notably, each branch is trained with an individual training pipeline. In the inference stage, the ultimate outcome is achieved by weighted summation of the prediction of the offline and online models.

#### 3.2.2. Decoupled Structure

**Figure 4:**
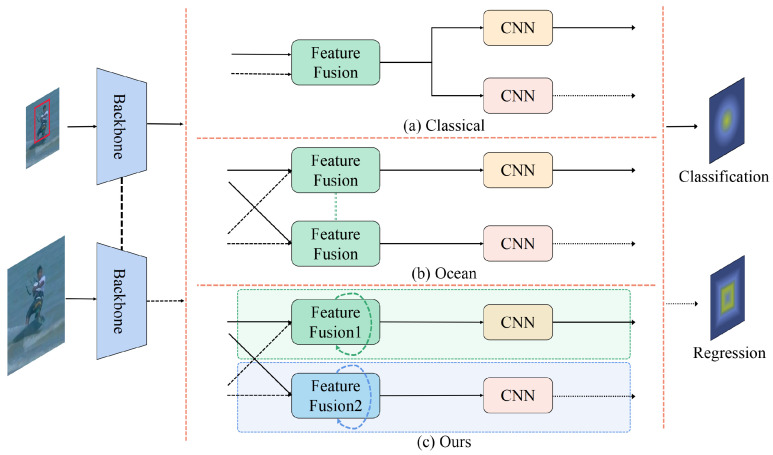
Comparison of decoupled structure of different trackers. (**a**) The decoupled structure in classic trackers, which shares the same feature correlation module in different branches. (**b**) The decoupled structure in Ocean, which uses two identical feature correlation modules with non-shared parameters. (**c**) The proposed decoupled structure. Unlike the previous two structures, we design an independent adaptive decoupled structure according to the characteristics of different branches, so that each branch learns the features related to the specific task independently and adaptively.

In the object detection field, TSD [7] spatially decouples the classification and localization tasks on the ROI features within the proposal regions to generate distinct classification and regression features. In the object tracking field, classic trackers share the same feature correlation module in different branches, as shown in Figure 4a. Ocean [8] designs a similar decoupling operation as TSD, as illustrated in Figure 4b. Ocean decouples template and search features through two identical cross-correlation operations to generate different response maps, which are then separately fed into the classification and regression branches for predictions. While generating unique input features may achieve a certain level of decoupling, the corresponding modules continue to remain identical, impeding their ability to cater to the distinct tasks of different branches. To address this issue, we propose a novel decoupled structure according to the characteristics of different branches, which utilizes distinct training pipelines, as depicted in Figure 4c. Importantly, our decoupled structure fully considers the task-specificity of different branches, which enables accurate tracking.

The classic CNN-based trackers perform cross-correlation operations on the template region *z* and the search region *x* to generate response maps *w*, which are then fed into the classification and regression branches to predict the class *Y* and the bounding box *B* of the object, respectively. The loss function of this process is described as follows:

(2) $$\begin{array}{c} L=L_{cls}\left(F_{1}\left(z,x\right)\|Y\right)+L_{reg}\left(F_{2}\left(z,x\right)\|B\right)\\ \left\{\begin{array}{c} F_{1}\left(\cdot \right)=\left\{\varphi \left(\cdot \right),g\left(\cdot \right),C\left(\cdot \right)\right\},\\ F_{2}\left(\cdot \right)=\left\{\varphi \left(\cdot \right),g\left(\cdot \right),R\left(\cdot \right)\right\} \end{array}\right. \end{array}$$

where φ(·) represents the feature extractor, g(·) denotes the cross-correlation operation, and C(·) and R(·) denote the classification function and regression function, respectively, which are used to generate predictions for the class and bounding box regressions.

Differing from conventional tracking methods, Ocean adopts two independent and identical cross-correlation operations to generate $w_{cls}$ and $w_{reg}$. However, our decoupled structure utilizes cross-correlation operations of the classification and regression branches to produce $w_{cls}^{D}$ and $w_{reg}^{D}$, which are further fed as inputs to the respective branches to mitigate the coupling between the branches. Additionally, our DLUT implements online template updating via the online queue branch, with the input features represented as $w_{queue}^{D}$. The final loss function can be expressed as follows:

(3) $$\begin{array}{c} L=L_{cls\;}^{D}\left(U_{cls}^{D}\left(z,x\right)\|Y\right)+L_{reg}^{D}\left(U_{reg}^{D}\left(z,x\right)\|B\right)+L_{queue}^{D}\left(U_{queue}^{D}\left(z,x\right)\|Y\right)\\ \left\{\begin{array}{c} U_{cls}^{D}\left(\cdot \right)=\left\{\varphi \left(\cdot \right),g_{c}^{D}\left(\cdot \right),C\left(\cdot \right)\right\},\\ U_{reg}^{D}\left(\cdot \right)=\left\{\varphi \left(\cdot \right),g_{r}^{D}\left(\cdot \right),R\left(\cdot \right)\right\},\\ U_{queue}^{D}\left(\cdot \right)=\left\{\varphi \left(\cdot \right),g_{q}^{D}\left(\cdot \right),Q\left(\cdot \right)\right\} \end{array}\right. \end{array}$$

where $g_{c}^{D}\left(\cdot \right)$, $g_{r}^{D}\left(\cdot \right)$, and $g_{q}^{D}\left(\cdot \right)$ denote decoupling-correlation operations designed to accommodate the characteristics of classification branch, regression branch, and online queue branch, respectively, and Q(·) is the prediction function of online queue branch.

### 3.3. Cross-Correlation Module

To accommodate the proposed unsupervised tracker based on the decoupled framework, we design three independent decoupling-correlation modules: the Pixel-FiLm-Depth-wise (PFD) module for the classification branch, the Pixel-Pair-Depth-wise (PPD) module for the regression branch, and the Pixel-Psa-Depth-wise (PLD) module for the online queue branch.

**Figure 5:**
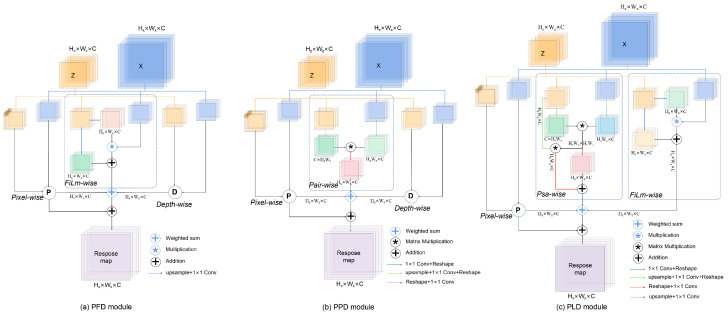
(**a**) Pixel-FiLm-Depth-wise (PFD) module corresponding to the classification branch. (**b**) Pixel-Pair-Depth-wise (PPD) module for the regression branch. (**c**) Pixel-Psa-Depth-wise (PLD) module for the online queue branch. Here, P and D represent Pixel-wise and Depth-wise operations, respectively.

The PFD module, as the decoupling-correlation module of the classification branch, comprises three distinct sub-modules. The left sub-module, called Pixel-wise (PW), employs small convolution kernels and diverse representations to enable the response map to contain more object edges and scale information. The right sub-module is Depth-wise (DW), which balances the number of parameters and performance while predicting multi-channel correlation features between the template and search region. The middle sub-module is FiLm-wise (FiLmW) [42], which helps the model to capture additional contextual information for more accurate foreground/background classification. We utilize PW and DW to compute multi-scale correlations between template features and search region features. Additionally, FiLmW establishes contextual relationships among multi-scale features to gather more information about target classification. The structure of the PFD module is depicted in Figure 5a, which is defined as follows:

(4) $$\begin{array}{c} R_{PFD}=PW\left(z,x\right)+\left(\omega_{1}\cdot PW\left(z,x\right)⊕\omega_{2}\cdot FiLmW\left(z,x\right)⊕\omega_{3}\cdot DW\left(z,x\right)\right)\\ FiLmW(z,x)=Conv(z)\cdot x+Conv(z) \end{array}$$

where $R_{PFD}$ is the response map generated by the PFD module. PW(·) and DW(·) denote Pixel-wise and Depth-wise, respectively. The symbol + represents the element-wise addition, · signifies the coefficient multiplication, and ⊕ denotes the weight summation. Conv(·) represents a 1 × 1 convolutional layer, and $\omega_{i}$ stands for the weight scaling factor, where i=1,2,3.

The PPD module, as the decoupling-correlation module of the regression branch, has a similar structure on both sides as the PFD module and is used to compute multi-scale correlations between template features and search region features. For the PPD module, we utilize Pair-wise (PairW) [9] to create feature pairs by combining search region features with template features. These pairs are reshaped and multiplied to generate response maps that measure the affinity between each unit and the candidate features. This helps our regression module to estimate the position of the target object accurately. Figure 5b illustrates the PPD module, which is defined as follows:

(5) RPPD=PW(z,x)+μ1·PW(z,x)⊕μ2·PairW(z,x)⊕μ3·DW(z,x)PairW(z,x)=Re(matmul(Re(z),Re(x)))

where $R_{PPD}$ is the response map generated by the PPD module. matmul(·) represents matrix multiplication, Re(·) stands for the Reshape(·) function, and $\mu_{i}$ is the weight scaling coefficient, where i=1,2,3.

The PLD module, as the decoupling-correlation module of the online queue branch, is used to calculate the correlation between multiple sets of template and search region features. It still retains PW and is located on the left side of the module. To improve the classification and regression performance, as well as to better accommodate correlation computations of the online queue branch, Psa-wise (PsaW) combines PairW in the PPD Module with the self-attention computation in Vision Transformer (ViT) [43]. Specifically, the PsaW module reshapes and multiplies feature pairs made up of templates and searches to obtain an affinity matrix between template features and search region features. Then, the affinity matrix is multiplied with the object features, that is, a self-attention operation is performed on the object features. The right side of the PLD module introduces FiLmW used in the PFD module. The network structure of the PLD module is illustrated in Figure 5c and is defined as follows:

(6) RPLD=PW(z,x)+θ1·PW(z,x)⊕θ2·PsaW(z,x)⊕θ3·FiLmW(z,x)PsaW(z,x)=Att(q,k,v)+Re(matmul(Re(z),Re(x)))

where $R_{PLD}$ is the response map generated by the PLD module. Att(·) represents a multi-head attention layer in ViT, and q=Conv(x), k=Conv(z), v=Conv(z), and $\theta_{i}$ are the weight scaling coefficients, where i=1,2,3.

### 3.4. Training the Network with Noisy Labels

After decoupling the network structure, our DLUT has the ability to differentiate between classification and regression tasks. Nevertheless, in practical implementation, our DLUT is reliant on pseudo-labels [3] generated by unsupervised optical flow for training. As a result of the lack of labels or the use of inadequate pseudo-labels, our unsupervised tracking training has its limitations. We believe that the quality of pseudo-labels is closely related to the efficacy of network training, so we further filter the training samples to select those that are beneficial for training and driving the network towards positive learning.

#### 3.4.1. Suppression-Ranking Strategy

**Figure 6:**
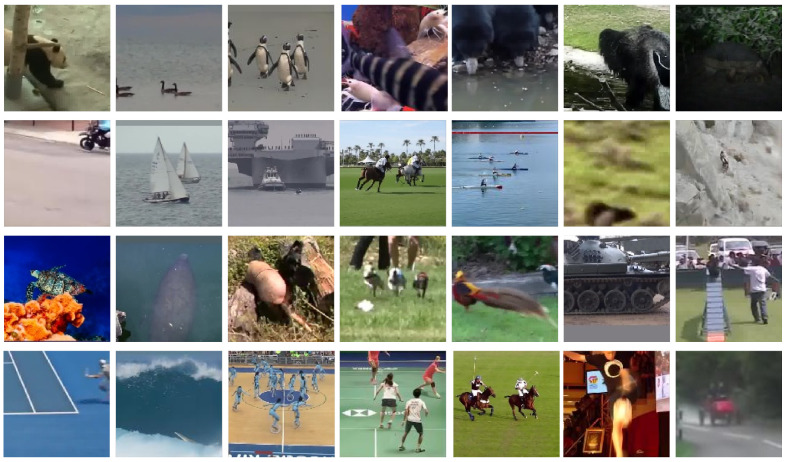
Visual examples of pseudo-labels generated by optical flow. Most pseudo-labels exhibit characteristics such as object loss, similar objects, illumination variations, and motion blur in the cropped templates.

Figure 6 shows visual examples of pseudo-labels generated by optical flow. The observation from Figure 6 indicates that the target templates contain a considerable amount of background noise. This situation creates an imbalance between positive and negative samples, which causes the domination of the easy-to-classify samples over the overall loss, thus dispersing the classifier’s attention. To tackle this issue, we investigate a Suppression-Ranking Strategy (SRS) for unsupervised learning. Specifically, inspired by Focal Loss [44] and RBO optimization [45], we further propose a novel classifier loss function that incorporates sample balance loss and classification-ranking loss, which not only suppresses extreme noise and balances the cross-entropy loss between positive and negative samples, prompting the tracker to focus more on challenging samples, but also highlights the importance of the foreground object.

To address the loss of positive and negative samples during training as well as to minimize the gradient contribution of easy-to-classify samples to our DLUT, we introduce focal loss as the sample balance loss:

(7) $$L_{balance}=-\rho \cdot {\left(1-y\right)}^{\delta }\log\left(y\right)-\left(1-\rho \right)\cdot y^{\delta }\times \log\left(1-y\right)$$

where $y\in \left[0,1\right]$ represents the prediction result, the weight parameter ρ balances positive and negative samples to adjust their importance, while the focusing parameter δ can be adjusted for weight on the hard sample. Increasing δ reduces the proportion of error from easy-to-classify samples in the overall loss. Sample balance loss is an updated cross-entropy loss function that applies a heavier penalty to easy-to-classify samples, thereby prioritizing the optimization of challenging samples and strengthening the model’s robustness.

Although sample balance loss remedied imbalances and diminished the loss of easy-to-classify samples, it inadequately emphasized the necessary object information, rendering the correct identification of foreground objects still problematic. To further highlight the object, we treat a classification loss function optimized by the RBO algorithm as the classification-ranking loss:

(8) $$L_{ranking}=\frac{1}{\beta }\cdot \log\left(1+\exp\left(\beta \cdot \left(P_{neg}-P_{pos}+\alpha \right)\right)\right)$$

where β controls the loss value, α represents a minimum threshold value, $P_{pos}$ stands for positive samples, and $P_{neg}$ stands for negative samples. classification-ranking loss aims to encourage the tracker to rank positive samples ahead of negative samples, enhancing target classification capability. Specifically, it utilizes a ranking-based approach to sort positive and negative samples and optimize the loss function based on their dissimilarity. This helps the tracker focus more on target classification while avoiding misclassifying background noise as a target.

Training with a single loss function from the above can effectively improve the performance of the network. However, using both loss functions concurrently in our classifier can render the resulting tracker susceptible to the influence of pseudo-label training. The classification loss is dominated by the training loss of extremely noisy samples, thereby compromising the training process despite the tracker’s ability to balance sample losses and rank positive and negative samples.

To minimize the impact of pseudo-labels on the training process, we employ a Primary Screening Operation (PSO) within the sample balance loss before applying classification-ranking loss. We specifically determine the loss value $L_{dec}^{\langle i,j\rangle }$ by utilizing Equation (9) to identify highly noisy samples with higher loss values. This guarantees that our classification-ranking loss can concentrate more accurately on target samples.

(9) $$L_{dec}^{\langle i,j\rangle }=-Y^{\langle i,j\rangle }\cdot \log\left({\tilde{y}}_{pred}^{\langle i,j\rangle }\right)-\left(1-Y^{\langle i,j\rangle }\right)\cdot \log\left(1-{\tilde{y}}_{pred}^{\langle i,j\rangle }\right)$$

where ${\tilde{y}}_{pred}^{\langle i,j\rangle }$ represents the predicted value for the *i*-th sample, while $Y^{\langle i,j\rangle }$ corresponds to the true label for that sample. The batch limit value $\varepsilon_{\mathrm{limit}}^{j}$ for batch is defined as:

(10) $$\varepsilon_{\mathrm{limit}}^{j}=2\cdot \left(\frac{1}{n}\cdot \sum_{i=0}^{n}L_{dec}^{\langle i,j\rangle }\right)-\min\left(L_{dec}^{\langle 0,j\rangle },L_{dec}^{\langle 1,j\rangle },\ldots ,L_{dec}^{\langle n,j\rangle }\right)$$

If the loss value of the *i*-th sample, denoted by $L_{dec}^{\langle i,j\rangle }$, exceeds $\varepsilon_{limit}^{j}$, the limit value of the corresponding batch *j*, its weight is assigned to ${\tilde{p}}_{\langle i,j\rangle }=0$. Otherwise, it is assigned to ${\tilde{p}}_{\langle i,j\rangle }=1$. It is worth noticing that the sample screening is restricted to the first *N* noisy samples in the batch that surpass the batch limit. Consequently, the improved sample balance loss is:

(11) $$L_{balance}^{pso}=-\left(\rho \cdot {\left(1-{\tilde{y}}_{\langle i,j\rangle }\right)}^{\delta }\log\left({\tilde{y}}_{\langle i,j\rangle }\right)+\left(1-\rho \right)\cdot {\tilde{y}}_{\langle i,j\rangle }^{\delta }\times \log\left(1-{\tilde{y}}_{\langle i,j\rangle }\right)\right)\cdot {\tilde{p}}_{\langle i,j\rangle }$$

Subsequent experiments demonstrate that the DLUT is able to efficiently synthesize the sample balance loss and classification-ranking loss through the application of PSO, resulting in an effective suppression-ranking training strategy. Detailed experiments can be found in our ablation study section.

In the end, the improved classification loss with the Suppression-Ranking Strategy is calculated as follows:

(12) $$L_{cls}=L_{balance\;}^{pso}+L_{ranking}$$

#### 3.4.2. Regression Loss

Training with pseudo-labels in the presence of background noise can result in significant errors in the original Intersection over Union (IOU), leading to a mismatch between predicted and actual boxes and rendering regression infeasible. Additionally, the strict IOU metric may not be ideal for the regression training of pseudo-labels because it could teach the tracker inaccurate regression box information. To address this issue, we assume that the regression branch generates the regression feature map R(i,j,:) and the regression targets for the four bounds of R(i,j,:) are represented as ${\tilde{o}}^{k}\left(i,j\right)\left|{}_{k=0,1,2,3}\right.$, we compute the regression loss by using

(13) $$L_{reg}=\frac{1}{\sum T\left({\tilde{o}}_{\left(i,j\right)}\right)}\sum_{i,j}T\left({\tilde{o}}_{\left(i,j\right)}\right)\cdot L_{DIoU}\left(R\left(i,j,:\right),{\tilde{o}}_{\left(i,j\right)}\right)$$

where $L_{DIoU}$ is the DIOU loss [46], $T\left(\cdot \right)$ is an indicator function, and if ${\tilde{o}}_{{}_{\left(i,j\right)}}^{k}\left|{}_{k=0,1,2,3}\right.>0$, then $T\left({\tilde{o}}_{\left(i,j\right)}\right)=1$, otherwise $T\left({\tilde{o}}_{\left(i,j\right)}\right)=0$.

#### 3.4.3. Centerness Loss

To address the creation of low-quality bounding boxes as a result of background noise, we employ a centerness branch training approach [47]. This method calculates the distance of each bounding box from the target center and uses that distance as a weight for adjusting the bounding box loss. The result is a decrease in weight for boxes that are far from the target center and an increase in weight for those that are closer. By focusing on the areas surrounding the target object, the tracker improves regression accuracy.

(14) $$L_{cen\;}=L_{centerness\;}$$

Due to the significant errors of the tracker in the initial stage of offline training, the training of the centerness branch currently holds no practical significance. Consequently, the gradient information of the centerness branch is only backpropagated during joint training in both offline and online stages.

### 3.5. Multi-Stage Training

**Training process.** To address the issue of inaccurately labeled pseudo-labels, similar to [3], we split the training process into two stages. In the first stage, only the offline module is used for training to acquire a naive Siamese tracker. The offline module is divided into classification and regression branches, which are used to handle classification and regression tasks, respectively. Our DLUT takes the template *z* and the search region *x* as the input. The classification branch is used to produce a 25 × 25 × 1 foreground/background classification response map *cls_pred*, while the regression branch is used to yield a 25 × 25 × 4 regression response map *reg_pred*. Furthermore, the centerness prediction head in the classification branch is used to generate a centerness response map *center-ness* with the same size as the classification response map. Since the loss for the first stage does not backpropagate the center-ness loss, it is defined as

(15) $$\begin{array}{c} L=\lambda_{1}\cdot L_{cls}+L_{reg} \end{array}$$

where $\lambda_{1}$ is the classification weight parameter coefficient.

The second stage consists of the offline module and the online module. Similar to the first stage, the offline module is composed of classification and regression branches. Yet, unlike the first stage, we backpropagate the centerness loss in this stage. For the online module, it first predicts the response map *queue_pred* between the template *z* and the interval search queue *x_queue*, along with the corresponding target bounding boxes *B*; then, it uses the features obtained by cropping *x_queue* with pseudo-labels as a new template, which is further used to predict the response map *queue_kernel* for *x_queue*; subsequently, the features of *queue_pred* and *queue_kernel* are fused as template features, and further combined with the depth features of the search region *x* to compute the feature correlation. For an interval search queue of length $N_{queue}$, the corresponding response map is denoted as $\left\{C_{corr\;}^{q}|1⩽q⩽N_{queue\;}\right\}$. Finally, the confidence strategy is leveraged to fuse $C_{corr\;}^{q}$ into the comprehensive response map $R_{queue\;}$.

(16) $$R_{queue}=Conv\left(\sum_{1\leq q\leq N_{queue}}Softmax\left(C_{conf}^{q}\right)⊙C_{val}^{q}\right)$$

where ⊙ denotes the Hadamard product, and Conv(·) is a 3 × 3 convolutional layer used to convert the interval search queue response map into a 25 × 25 × 1 comprehensive response map $R_{queue\;}$, which corresponds to the response mapping of the interval search queue over the search region *x*. Thus, the loss for the second stage is computed as:

(17) $$L=\lambda_{1}\cdot L_{cls}+L_{reg}+\lambda_{2}\cdot L_{queue}+L_{cen}$$

where $\lambda_{1}$ and $\lambda_{2}$ are weight coefficients, and $L_{queue}$ represents the online queue training loss. We utilize the proposed suppression-ranking training strategy and share the same set of labels with the classification loss.

**Template Update.** Classic trackers using fixed templates typically yield satisfactory tracking performance under standard conditions. Nonetheless, such trackers may lead to tracking drift or even tracking failure in situations where there exist challenges such as appearance changes, occlusion, and so on.

Our DLUT adopts a combination of online and offline training, yet updates the template in the online stage. The integrated response map *R* for online template update is represented as follows:

(18) $$R=\sigma \cdot R_{cls}+\left(1-\sigma \right)\cdot R_{queue}$$

where $R_{cls}$ denotes the classification response map generated from offline training, $R_{queue}$ denotes the integrated response map of the online queue generated from online training, and δ represents the weight coefficient.

## 4. Experiments

In this section, we first introduce the experimental setup and details. Then, we compare the proposed DLUT with state-of-the-art supervised and unsupervised trackers on numerous benchmark datasets, including OTB2015 [48], TrackingNet [49], LaSOT [50], TNL2K [51], and NFS [52]. Subsequently, we validate the effectiveness of the proposed modules through a series of ablation experiments. Finally, we visually present comparison results of various trackers on sequences from different datasets.

### 4.1. Experimental Setup and Details

We take the pseudo-labels generated by an unsupervised optical flow method [3] as the training dataset. ResNet50 [41] is used as the network backbone, and the features from the third layer are selected as the network input. To train the proposed DLUT, we conduct 45 epochs on two NVIDIA GeForce RTX3090 24GB GPUs by using the SGD [53] optimizer and warm-up training strategy. The initial 5 epochs are dedicated to offline training, where the learning rate increases from $2.5\times 10^{-3}$ to $5\times 10^{-3}$. The subsequent 40 epochs are dedicated to online training, where the learning rate decreases from $5\times 10^{-3}$ to $2\times 10^{-5}$ by using an exponentially decreasing function.

The scaling coefficients, namely $\omega_{i}$, $\mu_{i}$, and $\theta_{i}$, for three decoupling-correlation modules are all learnable parameters that can be adaptively adjusted to accommodate the different learning tasks. In the proposed Suppression-Ranking Strategy (SRS), the weight parameter ρ for positive samples is set to 0.25, while the adaptable focusing parameter δ is set to 2 in the sample balance loss (SBL). The sample screening operation processes a quantity of 10% noisy samples from the current batch. In the classification-ranking loss (CRL), the loss control value is set to β=4, with a minimum threshold value of α=0.5. During the preceding offline training, the weight coefficient is set to $\lambda_{1}=0.25$. During the subsequent online training, the queue length is set to $N_{queue}=3$. The weight coefficients adhere to the condition $\lambda_{2}=0.9-\lambda_{1}$. At this point, weight coefficient $\lambda_{1}=\left[0.35,0.325,0.3\right]$ gradually decreases during the training process. During template updating, the initial weight coefficients for calculating the comprehensive response map are set to $\sigma \in \left[0.7,0.6,0.5\right]$.

### 4.2. Comparison with State-of-the-Art Trackers

**OTB2015:** OTB2015 consists of 100 video sequences with eleven challenging attributes. We compare the proposed DLUT with state-of-the-art supervised and unsupervised trackers on OTB2015, and Table 1 presents quantitative results for different trackers. From Table 1, it is evident that our DLUT exhibits competitive tracking performance compared to supervised methods and even outperforms the supervised tracker SiamFC by approximately 4.6% in Success (Suc.) and 10% in Precision (Pre.). Moreover, compared to the two latest unsupervised trackers, although our DLUT outperforms ULAST in terms of both success and accuracy, it is inferior to GOT. The above results indicate that the proposed DLUT exhibits competitive performance on the OTB2015 dataset.

**TrackingNet:** The TrackingNet dataset is a large-scale tracking dataset, containing 511 video test sequences. An additional metric, i.e., Normalized Precision (NPre.), is introduced to evaluate the trackers. Table 1 also lists quantitative results of different trackers on TrackingNet. It can be seen from Table 1 that our DLUT also outperforms the supervised SiamFC, with 4.7%, 3.4%, and 0.7% improvement in Success, Precision, and Normalized Precision, respectively. Furthermore, compared with the latest unsupervised tracker, i.e., GOT, our DLUT also achieves the optimal Suc. (0.618), Pre. (0.567), and NPre. (0.700). This highlights the robust potential of unsupervised trackers on large-scale datasets.

**Figure 7:**
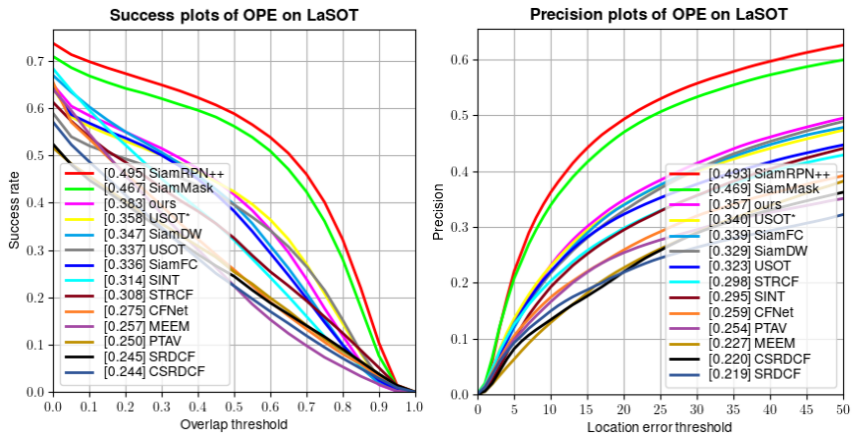
Success and Precision plots on LaSOT.

**LaSOT:** LaSOT is a dataset used to evaluate long-term tracking performance and consists of 280 video test sequences with an average length of over 2000 frames. Figure 7 illustrates that our DLUT achieves outstanding performance in Success (0.383) and Precision (0.357), surpassing the unsupervised tracker USOT* with a Success of 0.358 (2.5%↑) and a Precision of 0.340 (1.7%↑). Also, our tracker exhibits superior performance in comparison to some supervised trackers. These results indicate that the proposed DLUT is equally good at long-term tracking.

**NFS:** NFS is a dataset of 100 video sequences (380K frames) captured from real-world scenarios by high-speed cameras at low (30 FPS) and high (240 FPS) speeds. These sequences feature objects with fast motion, and our DLUT is evaluated on these sequences with different frame rates, including high frame rates (240 FPS), low frame rate with synthesized motion blur (30 FPS MB), and low frame rate without motion blur (30 FPS no MB). As presented in Table 2, our DLUT achieves the optimal performance in terms of AUC scores on sequences with high frame rates (240 FPS) and low frame rates (30 FPS no MB and 30 FPS MB). Specifically, the proposed DLUT outperforms the sub-optimal tracker MDNet by 0.8% and 3.1% in terms of AUC scores on sequences with low frame rates, respectively, and outperforms the sub-optimal tracker SiamFC by 3.2% in terms of AUC scores on sequences with high frame rate.

**Figure 8:**
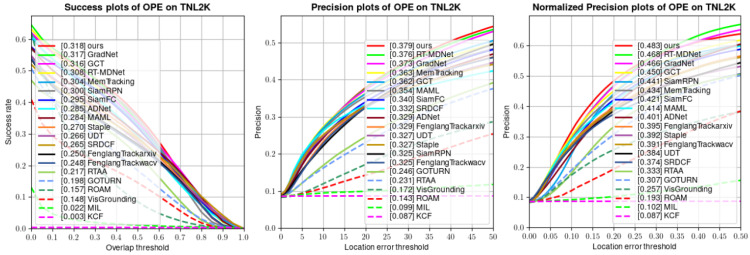
Success, Precision, and Normalized Precision plots on TNL2K.

**TNL2K:** TNL2K is a large-scale and high-quality multi-modal dataset containing 700 test video sequences. As depicted in Figure 8, the proposed DLUT is superior to all compared supervised and unsupervised trackers with the highest Success of 0.318 and Precision of 0.379. Specifically, compared to the unsupervised tracker UDT, the proposed DLUT gains 5.2%, 5.2%, and 9.9% improvement in Success, Precision, and Normalized Precision, respectively. The above results indicate that the proposed tracking paradigm possesses the capacity to handle diverse complex scenarios.

### 4.3. Ablation Experiments

In this section, we comprehensively validate the proposed decoupling-correlation modules and Suppression-Ranking Strategy (SRS) through a large number of experiments. In these ablation experiments, we compare and analyze the proposed methods on the OTB2015 dataset.

A series of experiments are conducted to explore the compatibility of the proposed three decoupling-correlation modules on different branches, as well as to verify the contribution of each module to our tracker, where the results are listed in Table 3. As can be seen from Experiments 1 to 3, the proposed PFD module provides the most significant improvements in Precision for the classification branch. Also, the proposed PFD module is the primary contributor to our tracker, followed by the PPD and PLD modules. From Experiments 4 to 6, we observe that the trackers using both decoupling-correlation modules are superior to the trackers using only a single one. Particularly, the tracker using both PFD and PPD modules yields the optimal results, with improvements in Precision of 0.3% and 2.7%, respectively, compared to the tracker using only the PFD module or the PPD module. In Experiment 7, all three modules are individually integrated into the respective branches of the tracker, which forms the proposed DLUT. Compared to the original tracker that serves as the reference group in Experiment 8, our DLUT produces gains of 4.4% and 7.5% in Success and Precision, respectively. Subsequently, we delve even further into the detailed analysis of the gains offered by these proposed modules.

As shown in Table 4, we design three decoupling-correlation modules by improving original Pixel-wise and Depth-wise operations. In this experiment, we separately replace the original operations with the designed modules, where the experimental results are shown in Table 4. It can be seen from Table 4 that the model with the proposed tracking branches achieves significant performance advancement in classification and regression tasks compared to the one with the original operations.

Specifically, the model with the proposed PFD module attains the greatest Success (63.6% and 67.0%) in dealing with illumination variation (IV) and low resolution (LR) challenges, as well as the highest Precision (87.0% and 97.6%) in handling deformation (DEF) and low resolution (LR) challenges, respectively. This suggests that the proposed PFD module has adaptability in circumstances where the object is influenced by illumination variation (IV) and deformation (DEF). At the same time, the model with the proposed PPD module achieves the best Precision (83.6%) in dealing with the background clutter (BC) challenge, while the model with the proposed PLD module achieves the best Success (52.9%) and Precision (74.2%) in coping with the out-of-view (OV) challenge. These results mean that our PPD and PLD modules assist the tracker in coping with situations where the object is influenced by the background clutter (BC) or out-of-view (OV), respectively. In contrast, by integrating the above three modules, our DLUT delivers optimal or sub-optimal results in various challenges as well as an overall improvement of 4.4% in Success and 7.5% in Precision. The noteworthy performance improvement indicates the efficacy of the designed decoupling-correlation modules in unlocking the tracking potential, facilitating the proposed DLUT to comprehensively tackle various real-world challenges.

**Figure 9:**
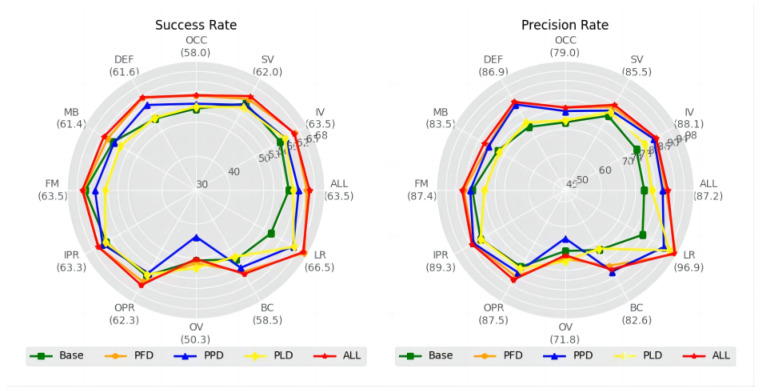
Attribute evaluation based on Success and Precision for the proposed decoupling-correlation modules (i.e., PFD, PPD, PLD) on the OTB2015 dataset with different attributes.

**Figure 10:**
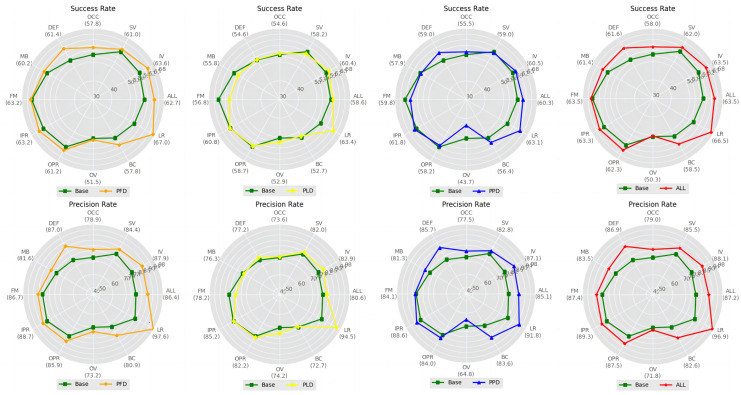
Visualization of Success (**first row**) and Precision (**second row**) for the proposed decoupling-correlation modules (i.e., PFD, PPD, PLD) of the classification branch, regression branch, and online queue branch on the OTB2015 dataset with different attributes.

As illustrated in Figure 9 and Figure 10, we can visually observe that the models with a single decoupling-correlation module experience a noticeable improvement in various attributes. The proposed DLUT with three decoupling-correlation modules achieves optimal performance across all attributes and produces significant enhancements in various scenarios.

Table 5 depicts the comparison of quantitative metrics for trackers using different loss functions. In Experiments 1 and 2, sample balance loss (SBL), and classification-ranking loss (CRL) are, individually, used to substitute the original cross-entropy loss (CEL) used in the baseline tracker in Experiment 7. As shown in Table 5, the models using only SBL or CRL yield 1.2% and 2.2% improvements in Success over the baseline tracker, respectively. However, the model using both SBL and CRL decreases significantly in both overall Success and Precision in Experiment 3. In Experiment 4, the primary screening operation is executed on the SBL function based on Experiment 3, that is, the tracker is trained with the Suppression-Ranking Strategy (SRS). Such a tracker improves the overall Success and Precision by 3.1% and 4%, respectively, and achieves optimal performance due to the suppression of extreme noise. Additionally, from Experiments 5–6, it can be observed that the primary screening operation is detrimental to the tracking performance whether it is introduced in CRL alone or both SBL and CRL. This is because the primary screening operation suppresses the contribution of positive samples, resulting in the foreground object being interfered with by noise. We attribute the above issues to the fact that the primary screening operation suppresses positive samples, resulting in the exclusion of foreground objects being mistakenly associated with the background. As a result, our primary screening operation is only integrated into the SBL function to suppress extreme noise.

To further validate the effectiveness of the proposed decoupled structure and Suppression-Ranking Strategy (SRS), we test the quantitative metrics of the trackers using the decoupling-correlation modules and/or the SRS separately, and the results are presented in Table 6. As can be seen from Table 6, the tracker only using the proposed decoupling-correlation modules outperforms the baseline tracker by 2.7% and 5.7% in Success and Precision, respectively, while the tracker only using the proposed SRS also outperforms the baseline tracker by 1.4% and 2.2% in Success and Precision, respectively. This further suggests the effectiveness of both the proposed decoupled structure and SRS. In contrast, the tracker using both the decoupling-correlation modules and the SRS strategy yields the optimal quantitative metrics. Compared to the baseline tracker, it improves by 5.8% and 9.7% in Success and Precision, respectively.

As shown in Table 7, the performance of our tracker is not significantly improved or becomes even worse in some cases when the centerness branch is only used in the first stage or both stages during training. In contrast, leveraging the centerness branch in the second stage yields the most favorable outcomes. Consequently, we only use the centerness branch for training in the second stage.

### 4.4. Qualitative Analysis

**Figure 11:**
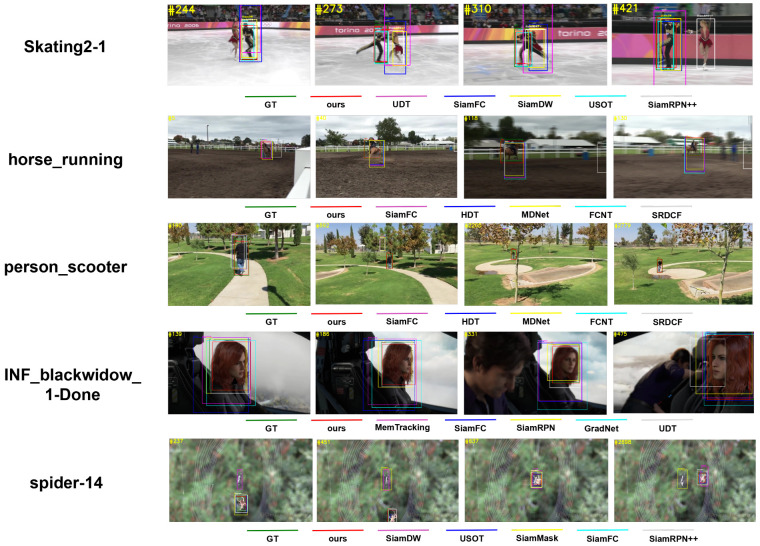
Comparison of qualitative results for various supervised trackers and unsupervised trackers (i.e., UDT, USOT). Tracking results of various trackers on respective sequences are marked with different color rectangles, and the frame numbers are shown in the top left corner of each frame. These results validate the robustness of the tracker against a variety of challenges, including fast motion, scale variation, motion blur, occlusion, and out-of-view, in both short-term and long-term scenarios. Notably, our DLUT even outperforms supervised trackers in some cases.

Figure 11 illustrates qualitative results for the proposed DLUT and various state-of-the-art supervised and unsupervised trackers on five sequences, including Skating2-1 (first row), horse_running (second row), person_scooter (third row), INF_blackwidow_1-Done (fourth row), and spider-14 (fifth row), from each benchmark dataset mentioned above, where the green box represents the ground-truth GT.

In Figure 11, the top row shows qualitative results of different trackers on the Skating2-1 sequence. During frame 244 without occlusion, most trackers successfully locate the target male skater. However, the female skater occludes the target in frame 273, and their positions are then quickly switched. During this process, only USOT and our DLUT remain unaltered and precisely track the male skater. By frame 310, most trackers begin to fail to consistently track the object. At frame 421, SiamRPN++ has lost the object, and the other compared trackers are significantly affected by high-speed motion and occlusion. However, our DLUT is consistently tracking the object.

In Figure 11, the second and third rows present comparison results of various trackers on the horse_running and person_scooter sequences, respectively. These two datasets contain objects with fast motion in real-world scenarios captured with high-speed cameras, providing an effective evaluation for the tracker to handle challenges, such as high-speed motion. In the horse racing scene shown in the second row, most trackers fail to capture the accurate position and scale of the jockeys as they move with speed. Between frames 118 and 130, the background blurs significantly, indicating a challenge for object tracking. Although SiamFC and MDNet are able to determine the jockey’s position, accurately estimating the target scale remains challenging. In contrast, our DLUT estimates the position and scale of the target continuously, facilitating robust tracking. The third row presents a person navigating a circular track while confronting challenges such as scale variation and partial occlusion, occurring between frames 892 and 2205. Among the above trackers, only DLUT maintains stable tracking throughout the whole process, including accurate scale estimation and target localization.

In Figure 11, the fourth row illustrates the qualitative results of different trackers on the INF_blackwidow_1-Done sequence. Most trackers fail to estimate the accurate target scale early due to appearance variations and complex backgrounds. The target is gradually blurred from frame 186 to 331, and the performance of most trackers is not affected. Nevertheless, at frame 475, most trackers fail to regress to the correct scale due to an abrupt change in the target position, yet our DLUT is capable of accurately regressing to the target position.

In Figure 11, the fifth row depicts the comparison results of various trackers on the spider-14 sequence. SiamDW and SiamMask [68] struggle to accurately locate the target due to the complex background. Starting at frame 451, the object goes out of view and then reappears; most trackers successfully relocate the object during this process. However, during frames 637 to 2698, the small scale and fast motion of the object prevent most trackers from accurately regressing to the target scale in real-time and only our DLUT achieves long-term, stable, and accurate tracking under such complex conditions.

Table 8 lists the comparison of the Success metric for various supervised and unsupervised trackers on the above five sequences. As can be seen from Table 8, the proposed DLUT outperforms supervised trackers across all test sequences.

Specifically, on the Skating2-1 sequence, our DLUT achieves a success rate of 0.595, substantially surpassing SiamFC. This indicates that our DLUT exhibits excellent performance in capturing the targets with dynamic and fast motion. On the horse_running sequence, our DLUT outperforms both MDNet and SiamFC with a score of 0.679, demonstrating that our tracker can handle the fast motion challenge. Similarly, on the person_scooter sequence, our DLUT maintains its lead with a score of 0.697, while the supervised tracker HDT scored 0.400. This highlights the adaptability of our DLUT to scale variations. On the sequence INF_blackwidow_1-Done, our DLUT gains a success rate of 0.732, still indicating its excellent performance. GradNet and MemTracking, as supervised trackers, also perform commendably with scores of 0.515 and 0.551, respectively, but are still inferior to our DLUT. On the spider-14 sequence, our DLUT obtains a score of 0.631, outperforming SiamMask at 0.605 and SiamRPN++ at 0.463. This indicates the effectiveness of the proposed unsupervised method in maintaining consistent tracking performance. Consequently, the above results highlight the superior performance of the proposed DLUT in various complex tracking scenarios.

## 5. Conclusions

To tackle the issue of high coupling among different branches in unsupervised trackers, we propose a decoupled learning-based unsupervised tracker (DLUT). The limitation of traditional unsupervised trackers lies in the inability to train different branches effectively. To this end, we develop a decoupled structure-based tracking framework that adapts to the specificity of different branches, thereby facilitating adequate training of each branch. Subsequently, to accommodate the decoupled framework, we design independent adaptive decoupling-correlation modules for different branches. These modules are capable of adjusting adaptively according to the characteristics of different branches during the training process. Furthermore, we present a Suppression-Ranking Strategy based on unsupervised training to adapt to the environment of pseudo-label training. This strategy not only balances the ratio of positive and negative samples but also emphasizes the weights of positive samples, thus facilitating our DLUT to train efficiently. Extensive experimental results validate that our DLUT is superior to state-of-the-art unsupervised trackers and achieves comparable performance to supervised trackers.

## Figures and Tables

**Table 1:** We compared the proposed DLUT with state-of-the-art supervised and unsupervised trackers on the OTB2015 and TrackingNet datasets. “Unsupervised” indicates whether a tracker belongs to an unsupervised one. In the case of unsupervised trackers, the best and second-best metrics are highlighted in red and blue, respectively.

Tracker	Unsupervised	OTB2015		TrackingNet		
		Suc.	Pre.	Suc.	Pre.	NPre.
SiamRPN++ [39]	No	0.695	0.906	0.694	0.800	0.733
SiamDW [54]	No	0.670	0.892	-	-	-
SiamRPN [11]	No	0.637	0.851	-	-	-
MDNet [55]	No	0.660	0.885	0.565	0.705	0.606
DCFNet [30]	No	0.580	0.769	0.533	0.654	0.578
SiamFC [10]	No	0.586	0.772	0.533	0.663	0.571
SRDCF [56]	No	0.598	0.789	0.455	0.573	0.521
CFNet [57]	No	0.568	0.778	0.533	0.654	0.578
Staple [58]	No	0.578	0.783	0.470	0.603	0.528
KCF [59]	Yes	0.485	0.696	0.419	0.546	0.447
DSST [60]	Yes	0.518	0.689	0.460	0.588	0.464
LUDT [61]	Yes	0.602	0.769	0.469	0.593	0.543
LUDT+ [61]	Yes	0.639	0.843	0.495	0.633	0.563
PUL [34]	Yes	0.584	-	0.485	0.630	0.546
USOT [3]	Yes	0.589	0.806	0.551	0.682	0.599
USOT * [3]	Yes	0.574	0.775	0.566	0.691	0.615
ULAST [4]	Yes	0.610	0.811	-	-	-
GOT [35]	Yes	0.654	0.876	0.526	-	0.563
ours	Yes	0.632	0.872	0.567	0.700	0.618

USOT * represents the version of the USOT tracker that has been pre-trained on ImageNet with supervision.

**Table 2:** Comparison of the AUC metric for different trackers on the NFS dataset under three tracking scenarios, including high-frame-rate tracking (240 FPS), low-frame-rate tracking with synthesized motion blur (30 FPS MB), and low-frame-rate tracking without motion blur (30 FPS no MB). The results are reported in terms of the AUC metric. The best and second-best metrics are highlighted in red and blue, respectively.

Tracker	KCF [59]	LCT [62]	HCF [63]	DSST [60]	SAMF [64]	Staple [58]	BACF [65]	SRDCF [56]	FCNT [66]	HDT [67]	SiamFC [10]	MDNet [55]	Ours
**30 FPS no MB**	0.209	0.232	0.275	0.277	0.281	0.321	0.332	0.339	0.378	0.386	0.396	0.418	0.426
**30 FPS MB**	0.203	0.222	0.270	0.261	0.270	0.310	0.320	0.331	0.365	0.368	0.367	0.395	0.426
**240 FPS**	0.308	0.318	0.357	0.416	0.411	0.421	0.467	0.448	0.447	0.453	0.454	0.450	0.486

**Table 3:** Ablation study on the contribution of the proposed three decoupling-correlation modules (i.e., PFD, PPD, PLD) to the Success (Suc.) and Precision (Pre.) metrics. Notably, we perform this experiment by using the proposed SRS. The optimal Success and the optimal Precision are highlighted in red and blue, respectively.

Experiment ID	PFD	PPD	PLD	Suc./Pre.
1	✓			62.7/86.4
2		✓		60.3/85.1
3			✓	58.6/80.6
4	✓	✓		63.0/86.6
5	✓		✓	62.9/86.0
6		✓	✓	60.9/83.1
7	✓	✓	✓	63.2/87.2
8				58.8/79.7

**Table 4:** Comparison of the Success (Suc.) and Precision (Pre.) metrics of decoupling-correlation modules (i.e., PFD, PPD, PLD) designed for the classification branch, regression branch, and online queue branch on the OTB2015 dataset with 11 different attributes, including illumination variation (IV), scale variation (SV), occlusion (OCC), deformation (DEF), motion blur (MB), fast motion (FM), in-plane rotation (IPR), out-of-plane rotation (OPR), out-of-view (OV), background clutter (BC), and low resolution (LR). For each attribute, the optimal Success and the optimal Precision are highlighted in red and blue, respectively.

	Overall	IV	SV	OCC	DEF	MB	FM	IPR	OPR	OV	BC	LR
**PFD**	62.7/86.4	63.6/87.9	61.0/84.4	57.8/78.9	61.4/87.0	60.2/81.6	63.2/86.7	63.2/88.7	61.2/85.9	51.5/73.2	57.8/80.9	67.0/97.6
**PPD**	60.3/85.1	60.5/87.1	59.0/82.8	55.5/77.5	59.0/85.7	57.9/81.3	59.8/84.1	61.8/88.6	58.2/84.0	43.7/64.8	56.4/83.6	63.1/91.8
**PLD**	58.6/80.6	60.4/82.9	58.2/82.0	54.6/73.6	54.6/77.2	55.8/76.3	56.8/78.2	60.8/85.2	58.7/82.2	52.9/74.2	52.7/72.7	63.4/94.5
**ALL**	63.2/87.2	63.5/88.1	62.0/85.5	58.0/79.0	61.6/86.9	61.4/83.5	63.5/87.4	63.3/89.3	62.3/87.5	50.3/71.8	58.5/82.6	66.5/96.9
**Base**	58.8/79.7	58.7/78.7	59.5/80.3	54.0/72.9	54.3/75.0	58.3/77.2	62.7/83.2	60.6/85.2	59.2/81.4	50.7/70.0	53.6/73.3	55.5/81.7

**Table 5:** Comparison of the Success (Suc.) and Precision (Pre.) metrics for the proposed Suppression-Ranking Strategy (SRS), sample balance loss (SBL), and classification-ranking loss (CRL), where Primary Screening Operation (PSO) is the crucial component of SRS. Notably, we conducted this experiment with the proposed decoupling-correlation modules. The optimal Success and the optimal Precision are highlighted in red and blue, respectively.

Experiment ID	SBL	CRL	PSO	Suc.	Pre.
1	✓			61.3	83.6
2		✓		62.3	85.2
3	✓	✓		60.7	82.3
4	✓		✓	63.2	87.2
5		✓	✓	61.7	84.8
6	✓	✓	✓	61.5	83.9
7				60.1	83.2

**Table 6:** Comparison of the Success (Suc.) and Precision (Pre.) metrics for the proposed decoupling-correlation modules and Suppression-Ranking Strategy (SRS). The optimal Success and the optimal Precision are highlighted in red and blue, respectively.

Decoupled-Correlation Modules	SRS	Suc.	Pre.
✓		60.1	83.2
	✓	58.8	79.7
✓	✓	63.2	87.2
		57.4	77.5

**Table 7:** Comparison of the Success (Suc.) and Precision (Pre.) metrics of the centerness branch in different stages (Stage 1 and Stage 2). The optimal Success and the optimal Precision are highlighted in red and blue, respectively.

Offline (St.1)	Offline+Online (St.2)	Suc.	Pre.
✓		62.1	86.3
	✓	63.2	87.2
✓	✓	62.5	86.4
		62.4	86.6

**Table 8:** Comparison of the Success metric for various supervised and unsupervised trackers on five sequences from different benchmark datasets. “Unsupervised” indicates whether a tracker belongs to an unsupervised one. The best and second-best metrics are highlighted in red and blue, respectively.

Tracker	SiamFC	MDNet	HDT	SRDCF	FCNT	GradNet	Mem- Tracking	SiamRPN	SiamMask	SiamRPN ++	SiamDW	UDT	USOT	Ours
Unsupervised	No	No	No	No	No	No	No	No	No	No	No	Yes	Yes	Yes
Skating2-1	0.335	-	-	-	-	-	-	-	-	0.454	0.365	0.327	0.560	0.595
horse_running	0.495	0.508	0.492	0.031	0.492	-	-	-	-	-	-	-	-	0.679
person_scooter	0.279	0.266	0.276	0.400	0.277	-	-	-	-	-	-	-	-	0.697
INF_blackwidow _1-Done	0.545	-	-	-	-	0.515	0.551	0.717	-	-	-	0.662	-	0.732
spider-14	0.063	-	-	-	-	-	-	-	0.401	0.605	0.463	-	0.641	0.631

## Data Availability

Data sharing is not applicable to this article as no datasets were generated or analyzed during the current study. However, if you need the code of the relevant module you can contact the author by email: faith2916290349@163.com.

## Abbreviations

The following abbreviations are used in this manuscript:

DLUT	Decoupled Learning-based Unsupervised Tracker
PFD	Pixel-FiLm-Depth-wise
PPD	Pixel-Pair-Depth-wise
PLD	Pixel-Psa-Depth-wise
SRS	Suppression-Ranking Strategy
SBL	Sample Balance Loss
CRL	Classification-Ranking Loss
DIoU	Distance-Intersection over Union
GPU	Graphic Processing Unit
SGD	Stochastic Gradient Descent
AUC	Area under curve
Suc	Success
Pre	Precision
NPre	Normalized Precision
GT	Ground Truth
